# Expression of the Phosphatase Ppef2 Controls Survival and Function of CD8^+^ Dendritic Cells

**DOI:** 10.3389/fimmu.2019.00222

**Published:** 2019-02-12

**Authors:** Markus Zwick, Thomas Ulas, Yi-Li Cho, Christine Ried, Leonie Grosse, Charlotte Simon, Caroline Bernhard, Dirk H. Busch, Joachim L. Schultze, Veit R. Buchholz, Susanne Stutte, Thomas Brocker

**Affiliations:** ^1^Faculty of Medicine, Biomedical Center (BMC), Institute for Immunology, LMU Munich, Planegg-Martinsried, Germany; ^2^Life and Medical Sciences Institute, Bonn, Germany; ^3^Institute for Medical Microbiology, Immunology and Hygiene, Technische Universität München, Munich, Germany; ^4^PRECISE—Platform for Single Cell Genomics and Epigenomics at the German Center for Neurodegenerative Diseases (DZNE) and the University of Bonn, Bonn, Germany

**Keywords:** apoptosis, CD8 T cell priming, cross-presentation, dendritic cells, immune homeostasis, DC-maturation

## Abstract

Apoptotic cell death of Dendritic cells (DCs) is critical for immune homeostasis. Although intrinsic mechanisms controlling DC death have not been fully characterized up to now, experimentally enforced inhibition of DC-death causes various autoimmune diseases in model systems. We have generated mice deficient for *Protein Phosphatase with EF-Hands 2* (Ppef2), which is selectively expressed in CD8^+^ DCs, but not in other related DC subtypes such as tissue CD103^+^ DCs. Ppef2 is down-regulated rapidly upon maturation of DCs by toll-like receptor stimuli, but not upon triggering of CD40. Ppef2-deficient CD8^+^ DCs accumulate the pro-apoptotic *Bcl-2-like protein 11* (Bim) and show increased apoptosis and reduced competitve repopulation capacities. Furthermore, *Ppef2*^−/−^ CD8^+^ DCs have strongly diminished antigen presentation capacities *in vivo*, as CD8^+^ T cells primed by *Ppef2*^−/−^ CD8^+^ DCs undergo reduced expansion. In conclusion, our data suggests that Ppef2 is crucial to support survival of immature CD8^+^ DCs, while Ppef2 down-regulation during DC-maturation limits T cell responses.

## Introduction

DCs are major antigen-presenting cells (APC) located in tissues and lymphoid organs, where they integrate environmental signals to initiate either immunity or tolerance. They express receptors for pathogen associated molecular patterns allowing early recognition of microbial intruders. By conveying this information to T lymphocytes, DCs are linking innate and adaptive immunity.

Classical DCs (cDC) develop from committed DC precursors (pre-DC) in the bone marrow (BM), which seed tissues and develop into different DC subsets (1, 2). As suggested recently, DCs can be grouped into cDC1 and cDC2 subsets, according to their developmental origin, functional properties and location (3). According to this nomenclature cDC1 express markers such as CD8 and CD103, while cDC2 express CD4 and CD11b. cDC1 are superior to present antigen via MHCI to CD8^+^ T cells (4–6), while cDC2 are better equipped to present antigen via MHCII to CD4 T cells (4). In addition, cDC1 are able to efficiently load exogenous antigen onto MHCI for cross-presentation to CD8^+^ T cells (7). More recently it was shown that cDC1 have the specific and non-redundant role to optimize CD8^+^ T cell responses during a later step in T cell priming (8). Several mutant mice have been generated to study the functions of cDC1 or cDC2 *in vivo* (2, 9). However, mice with mutations that discriminate cDC1 in different locations, such as splenic CD8^+^ cDC1 or intestinal CD103^+^ cDC1, but not both do not yet exist.

The maintenance of DC populations relies on constant replenishment by blood-borne precursors (10, 11) and *in situ* cell division with 5% of lymphoid organ resident DCs undergoing cell division at any given time (12). The importance of tightly controlled DC-numbers becomes obvious when the system is disturbed artificially. Inhibition of DC apoptosis by interfering either with caspases (13), pro-apoptotic Bim (14) or cell-death inducing Fas (15) *in vivo* caused DC-accumulation and autoimmunity. Similarly, artificial prolongation of DC-lifespans by Akt mutants (16) or overexpression of anti-apoptotic Bcl-2 (17) enhanced immunogenicity of DCs. However, mechanisms naturally regulating the DC lifespan are less well-described. DC-activation by lipopolysaccharide (LPS) induces apoptosis by CD14-mediated NFAT activation (18) and down-regulation of Bcl-2 (19). Also killing of DCs by primed cytotoxic T cells (CTL) has been described (20), a mechanism which was observed for both, CD103^+^ and CD11b^+^ DC (21). Ligands of the tumor necrosis factor superfamily bind to CD40 (22) or TRANCE (23) on DCs to prolong their survival (24). However, to the best of our knowledge other intrinsic DC life-cycle regulatory mechanisms are not known.

Previously, we characterized the promoter regions of CD11c and DC-STAMP, two DC-specific markers, and identified an evolutionary conserved promoter framework, which also controls expression of Ppef2 (25). Ppef2 is a poorly characterized phosphatase with three EF-hands typical for calcium-binding proteins and an IQ motif (26). In mice, Ppef2 is strongly expressed in the retina, but Ppef2 deficiency did not cause retinal degeneration (27), while Ppef2-orthologs prevent retinal degeneration in *Drosophila* (28). Besides the Ca^2+^- binding of Ppef2 (rdgC) in *C. elegans* (29) or the Calmodulin-binding of Ppef2 in human cells (30), it has been speculated that Ppef2 would be involved in stress-protective responses and could possibly positively regulate cell survival, growth, proliferation and oncogenesis as a “survival-phosphatase” (31, 32).

Here, we show that in the hematopoietic system of mice Ppef2 expression is confined to CD8^+^ DCs, but not tissue resident CD103^+^ DCs or other cells. Ppef2 is down-regulated rapidly after DC-activation with toll-like receptor (TLR) ligands, while DC-activation via CD40 did not alter Ppef2-levels. We generated Ppef2-deficient mice and show that splenic CD8^+^ cDC1 display increased apoptosis *in vitro* and *in vivo*. As a consequence, the *Ppef2*^−/−^ DCs displayed strongly reduced cross-presenting capacities *in vivo*. Our data identify Ppef2 as a molecular regulator for survival and function of CD8^+^ cDC1.

## Materials and Methods

### Mouse Strains

Alleles targeting Ppef2 were produced for the EUCOMM and EUCOMMTools projects by the Wellcome Trust Sanger Institute. JM8A3.N1 embryonic stem cells targeting the Ppef2 locus were purchased at EUCOMM and microinjection in C57BL/6 oocytes was performed by the Transgenic Core Facility at the Max-Planck-Institute for Molecular Cell Biology and Genetics in Dresden, Germany. Chimerism was determined by coat color, verified by genotyping and chimeric mice were further crossed with C57BL/6 mice. Mice were analyzed in sex and age-matched groups with 8–13 weeks of age. Animal experiment permission was granted by animal ethics committee Regierung von Oberbayern, Munich, Germany. *Ppef2*^−/−^, Ppef2^+/+^, and OT-I mice were bred and maintained at the animal facility of the Institute for Immunology, Ludwig-Maximilians-Universitaet München. H2-K^bm1^ mice were bred and kept in the Institut für Medizinische Mikrobiologie, Immunologie und Hygiene at the TU Munich.

### *In vitro* Cell Cultures

For GM-CSF BMDC cultures 10^7^ cells were plated in 10 ml of GM-CSF containing medium (20 ng/ml GM-CSF). At day 3 of the culture, cells were harvested with Trypsin and again plated at a density of 5 × 10^6^ cells in GM-CSF medium. For analysis, cells were harvested at day 8 of the culture with cold PBS. For Flt3L cultures bone marrow cells (1.5 × 10^6^/ml) were cultured Flt3L medium (200 ng/ml Flt3L; 500 ml RPMI1640, 10% FCS, 0.5 μM 2-mercaptoethanol,100 U/ml Penicillin, 100 μg/ml streptomycin, 0.1 mM nonessential aminoacids, 1% Glutamax, 1 mM Sodium Pyruvat) and harvested at day 8 for analysis. Mature BMDCs were obtained by stimulating overnight with 2 μg/ml lipopolysaccharide (LPS, Sigma-Aldrich), 1 μg/ml Flagellin, 2.5 μg/ml Poly I:C, 1 μg/ml Pam3CSK4, 2.5 μg/ml CLO97, or 100 μg/ml anti-CD40, respectively.

### Bone Marrow Chimeras

To generate bone marrow-chimeras recipient mice were irradiated with two split doses of 550 rad using a Cesium source (Gammacell 40, AECl,Mississauga, Canada). Irradiated animals were reconstituted with 5 × 10^6^ BM cells, 1:1 mixed from CD45.1^+^ and CD45.2^+^ BM. To prevent infection, animals received 1.2 g/l neomycin in water *ad libitum* for 4 weeks. Animals were analyzed 8–10 weeks after reconstitution.

### Flow Cytometry Analysis

Where possible, 2 × 10^6^ cells were used for every staining with titered antibodies in PBS containing 2% FCS and 0.01% NaN3 (fluorescence-activated cell sorting (FACS) buffer) and staining was carried out for 20 min at 4°C in the dark. Cells were washed once and used for direct acquisition on BD FACSCanto II. Dead cells were excluded using Aqua LIVE/DEAD Fixable Aqua DeadCell Stain Kit (Invitrogen, TermoFischer, Cat: L34957) or Zombie Aqua Fixable Viability Kit (BioLegend, Cat: 423102).

For the staining of cleaved caspase-3 cells were washed once and then resuspended in 200 μl 4% PFA for 15 min at room temperature in the dark. After washing twice with 1x fixation/permeabilisation buffer (BD) cells were blocked for 10 min with an anti CD16/32 antibody (Fc block, clone 2.4G2, BD) and 0.1% goat serum at 4°C. After 10 min of blocking, 100 μl anti cleaved caspase-3 antibody (clone D3E9, Cell Signaling) was added and cells were incubated for 30 min at 4°C. Unbound antibody was washed away and cells were incubated with 100 μl fluorochrome conjugated secondary antibody (goat anti-rabbit, Life Technologies) at 4°C for 30 min. Afterwards, cells were washed once and acquired at the FACS.

### Single-Cell Preparation

Single-cell suspensions of spleens, lymph nodes and thymi were prepared by digesting with DNAse I (0.2 mg/ml) and Liberase (0.65 Wuensch units/ml, both Roche) for 30 min at 37°C. Tissues were passed through a 100 μm cell strainer, washed once with cold PBS and red blood cells were lysed. A single-cell suspension of lung cells was performed like for the spleen, lymph nodes and thymi after flushing out the blood of the lung by disruption of the aorta followed by injection of cold PBS in the right ventricle. For analysis of dermal and epidermal cells ears were removed. The dorsal and ventral layers were separated with fine forceps and incubated dermal face down in 2 U/ml of Dispase II (Roche) in HBSS in 24 well-plate for 90 min at 37°C. Skin sheets were separated into dermis and epidermis with fine forceps in cold PBS. Epidermal sheets were further incubated for 2 h at 37°C in HBSS with 157 U/ml of collagenase IV (157 Wuensch units per ml, Worthington) and 10% FCS in 24 well-plate. Dermal sheets were digested for 2 h at 37°C in a solution containing 0.5 mg/ml of DNase I, 2.7 mg/ml of Collagenase XI, 27 μg/ml of Hylaronase VI and 10 mM of Hepes in RPMI. The suspensions were passed through a cell strainer, washed and counted. Isolation of liver cells was carried out by mashing the organ through a 100 μm mesh in PBS. Afterwards, mononuclear cells were enriched applying a gradient using Lymphoprep (STEMCELL Technologies). Hepatocytes and other cells were cleared out and positively enriched cell fraction was further used. To analyze cells from the lamina propria, colon was taken from a mouse, fecal content removed, the colon opened longitudinally and cut into ca. 5 mm big pieces. The pieces were then incubated with Hank's balanced salt solution (HBSS)-EDTA for 10 min on a shaker at 37°C, the supernatant containing epithelial cells was discarded and gut parts were washed twice with ice cold PBS. Afterwards the colon was digested once for 30 min and then twice for 20 min with a mixture of Collagenase IV (157 Wuensch units per ml, Worthington), DNAse I (0.2 mg/ml dissolved in PBS) and Liberase [0.65 Wuensch units per ml, both Roche, dissolved in Hank's Balanced Salt Solution with fetal calf serum (FCS)], the supernatant was collected after each digestion and the cells were washed once with PBS. Cells from all three digestions were combined and immune cells enriched using gradient centrifugation. For this, cells were resuspended in 40% Percoll and this solution was overlayed onto 80% Percoll solution. Centrifugation was carried out for 20 min at 1,800 r.p.m. and 4°C without break. Cells at the interphase were collected, washed once and used for further analysis.

### Transcriptional Analysis

Total RNA from sorted cells was isolated using TRIZOL and cDNA was generated using QuantiTect Reverse Transcription Kit (QIAGEN, Cat No: 205311). TaqMan PCR was performed using the Universal Probe Library Set mouse (Roche) according to manufacturer's instructions. Gene expression was normalized to HPRT expression. The following Primers were used: HPRT forward 5′-TCCTCCTCAGACCGCTTTT-3′, reverse 5′- CCTGGTTCATCATCGCTAATC-3′, probe #95; Ppef2 exon 2-3 forward 5′- AGGAGGCGATGTACCTGGA-3′, reverse 5′- CAAGGTAGCTGAAGAATTCATGG-3′, probe #56; Ppef2 exon 4-5 forward 5′-CTTCCTGACCATGCCACTG-3′, reverse 5′-TATCGAGCATGGAGCTGTTG-3′, probe #31; Ppef2 exon 11-12 forward 5′-TTCTGTCACAACCGCAAGG-3′, reverse 5′-TCTGTTGCTGCCAACTTCAT-3′, probe #16; Trim2 forward 5′-TTTCCATAATCACTCTGTCAAGGT-3′, reverse 5′-CCATTGGAGCCAAACTTCA-3′, probe #12; Dll4 forward 5′-AGGTGCCACTTCGGTTACAC-3′, reverse 5′-GGGAGAGCAAATGGCTGATA-3′, probe #106. When not stated otherwise primers used for the qPCR of Ppef2 included the primers ranging from exon 11–12. Methods for analyzing qPCR data including the ΔΔCt method for relative expression, as well as the 2-ΔCt method.

### Generation of cDNA Libraries

Total RNA was converted into libraries of double stranded cDNA molecules as a template for high throughput sequencing following the manufacturer's recommendations using the Illumina TruSeq RNA Sample Preparation Kit v2. Shortly, mRNA was purified from 100 ng of total RNA using poly-T oligo-attached magnetic25 beads. Fragmentation was carried out using divalent cations under elevated temperature in Illumina proprietary fragmentation buffer. First strand cDNA was synthesized using random oligonucleotides and SuperScript II (Invitrogen). Second strand cDNA synthesis was subsequently performed using DNA Polymerase I and RNase H. Remaining overhangs were converted into blunt ends via exonuclease/polymerase activities and enzymes were removed. After adenylation of 3′ ends of DNA fragments, Illumina indexed PE adapter oligonucleotides were ligated. DNA fragments with ligated adapter molecules were selectively enriched using Illumina PCR primer PE1.0 and PE2.0 in a 15 cycle PCR reaction. Size-selection and purification of cDNA fragments with preferentially 200–300 bp in length was performed using SPRIBeads (Beckman-Coulter). The size-distribution of cDNA libraries was measured using the high sensitivity D1000 assay on a 2,200 TapeStation instrument (Agilent). cDNA libraries were quantified using KAPA Library Quantification Kits (KapaBiosystems). After cluster generation on a cBot, a 50 bp single-end run was performed on an Illumina HiSeq1500.

### mRNA-Sequencing

After base calling and de-multiplexing using CASAVA version 1.8 the 50 bp single-end reads were aligned to the murine reference genome mm10 from UCSC by HISAT version 0.1.7-beta using the default parameters. After mapping of the reads to the genome, we imported the data into Partek Genomics Suite V6.6 (PGS) to quantify the number of reads mapped to each gene annotated in the RefSeq mm10 annotation downloaded in May 2015 resulting in 16,928 transcripts. These raw read counts were used as input to DESeq2 for calculation of normalized signal for each transcript using the default parameters. After DESeq2 normalization the normalized read counts were imported back into PGS and floored by setting all read counts to at least a read count of 1. Subsequent to flooring, all transcripts having a maximum over all groups means lower than 10 were removed. After dismissing the low expressed transcripts the data comprised of 10.722 transcripts. RNA-Seq data can be accessed in Gene Expression Omnibus under GSE (https://www.ncbi.nlm.nih.gov/geo/query/acc.cgi?acc=GSE98235).

In order to show whether the loss of Ppef2can be verified on the transcript level, read counts were visualized by IGV mapping the aligned reads against the mouse genome (mm10).

A one-way ANalysis Of VAriance (ANOVA) model was performed to calculate the differentially expressed genes (transcripts) between Ppef2^+/+^ and *Ppef2*^−/−^ cells using PGS. Differentially expressed genes were defined by a fold change (FC) > 2 or < −2 and a *p*-value < 0.01 resulting in 8 up-regulated and 13 down-regulated genes comparing *Ppef2*^−/−^ with Ppef2^+/+^. Visualization by volcano plot was performed using Prism v5.0c.

### Antibodies

Antibodies with the following specificities were used: CD3 (145-2C11, biotin, #553060), CD4 (H129.19, FITC,#553650; RM4-5, PerCP, #553052); CD16/32 (2.4G2, unlabeled, #553142); CD19 (1D3, APC, #550992; PE, #553786; Pe-Cy7, #552854; Biotin, #553784); CD43 (S7, Biotin, #553269), CD45 (30-F11, PerCP, BD, #557235), CD45R (RA3-6B2, PE, #553090), CD90.1 (OX-7, PerCP, #557266), CD103 (M290, PE, #557495), CD161 (PK136, PE, #553165; APC, #550627), CD172a (P84, APC, #560106), Gr1 (RB6-8C5, PE, #553128), Ly6C (AL-21, FITC,#553104; PE, #560592), Ly6G (1A8, APC, #560599), Vb5 (MR9-4, FITC, #553189) from Becton Dickinson. CD8 (53-6.7, Brilliant Violet, #100738), CD11c (N418, PeCy7, #117318), CD45 (30-F11, Brilliant Violet #103133), CD45R (#103240; RA3-6B2, PerCP); CD64 (X54-5/7.1, APC, #139305), CD86 (GL-1, PerCP, #105026), Dll4 (HMD4-1, PE, #130802), ESAM (1G8, PE, #12-5852-82), MHCII (M5/114.15.2, FITC, #107606; PerCP, #107624); SiglecH (551, APC, #129608) from BioLegend. CD3 (145-2C11, FITC, #11-0031-82; PE, #12-0031-82;); CD4 (GK1.5, PeCy7, #25-0041-81); CD8 (53-6.7, APC-Cy7, #47-0081-82; PeCy7, #25-0081-82), CD11b (M1/70, APC-Cy7, #47-0112-82), CD11c (N418, APC, #17-0114-82), CD45 (30-F11, PE, #12-0451-82), CD45.1 (A20, PE, #12-0453-83), CD45R (RA3-6B2, Brilliant Violet, #103240), CD90.1 (HIS51, APC, #17-0900-82), CD115 (AFS98, APC, #17-1152-82; AFS98, PeCy7, #25-1152-82), F4/80 (BM8, Pe-Cy7, #25-4801-82; PE, #12-4801-82), MHCII (M5/114.15.2, APC, #17-5321-82), SiglecH (eBio440c, PE, #12-0333-82; 551, PerCP, #129614), Va2 (B20.1, PE, #12-5812-82) from eBioscience. Cleaved caspase-3 (D3E9, unlabeled, #9579S) from Cell Signaling; goat anti-rabbit (PE, #P2771MP) from Life Technologies; Langerin (929F3.01, FITC, #DDX0362A488) from Dendritics.

### Measurement of β-Galactosidase (lacZ) Expression and Activity

Measurement of the expression and activity of the bacterial β-galactosidase gene (lacZ) was carried out using the FluoReporter lacZ Flow Cytometry Kit (Thermo Fisher Scientific Inc.) according to the manufacturers protocol. Therefore, the activity of the enzyme was measured by adding substrate [fluorescein di-V-galactoside (FDG)] for 2 min at 37°C and stopping of the reaction by addition of cold staining medium. Followed by normal surface antibody staining, samples were analyzed using a FACSCanto II.

### Cell Sorting

Cell sorting was either performed by staining the samples with fluorescently labeled antibodies and FACS-sorting the cells at a FACSAria or FACSFusion, or by staining the samples with magnetically labeled antibodies with MACS columns.

### T Cell Priming Assays

0.5 × 10^6^ purified OT-I cells were transferred intravenously into congenic mismatched Ppef2^+/+^ and *Ppef2*^−/−^ mice that received 100 μg grade IV OVA protein 1 day later i.v. *In vivo* T cell proliferation was analyzed 3 days after OVA injection staining for CD8 and CD90.1 or CD45.1, respectively. For immunization with anti-CD40 mAb, 100 μg FGK4.5 (BioXCell, West Lebanon, USA) were co-injected together with 30 μg OVA i.v.

For DC vaccinations, DCs were isolated from spleens of Ppef2^+/+^ and *Ppef2*^−/−^ mice as previously described (33, 34) CD11c^+^ GFP^high^ splenocytes were sorted on a MoFlo™ XDP cell sorter, using fluorochrome-labeled antibodies specific for CD3 (500A2), CD19 (1D3), CD11c (N418), and propidium iodide (PI, Invitrogen) for live-dead discrimination. Sorted DCs were then pulsed with 1 μg/ml OVA257 (SIINFEKL) peptide for 60 min (37°C) and washed three times with an excess volume of PBS. 1.5 × 10^5^ OVA257 pulsed DCs were injected i.v. and transferred into H-2K^bm1^ recipient mice intravenously. 24 h later 10^5^ H-2K^bm1+/+^ CD45.1^+^ naive CD8^+^CD44^lo^ OT-I cells were transferred and T cell expansion was measured 3 days later.

### RNA-Isolation

Spleen CD11c^+^MHCII^+^CD8^+^CD11b^−^ DCs were sorted on a FACSAria five independent times with three pooled Ppef2^+/+^ or *Ppef2*^−/−^ mice per sort. For RNA isolation 5 × 10^5^-6 × 10^6^ cells per sample were harvested, subsequently lysed in TRIzol (Invitrogen), and total RNA was extracted with the miRNeasy kit (Qiagen) according to the manufacturers protocol. The quality of the RNA was assessed by measuring the ratio of absorbance at 260 nm and 280 nm using a Nanodrop 2000 Spectrometer (Thermo Scientific) as well as by visualization of the integrity of the 28 and 18 S bands via a RNA analysis ScreenTape assay on a 2200 TapeStation instrument (Agilent).

### Western Blot Analysis

Proteins were separated by SDS-PAGE (15%). Cell lysates were quantified using the Quant-iT Protein Assay kit (Molecular Probes). Equal amounts of protein were loaded onto 15% SDS gels, and GAPDH (clone 14C10, Cell signaling) was used as a loading control. After transferring to a nitrocellulose membrane, proteins were incubated with primary antibodies against Bim (clone C43C5, Cell Signaling) or GAPDH, washed, and incubated with secondary HRP-labeled antibodies (Jackson Immuno-Research Laboratories). Then, membranes were visualized using luminescent substrate ECL (GE Healthcare). Western blot bands were quantified using ImageJ software (National Institutes of Health).

### Statistics

For absolute cell numbers the percentage of living cells of a given subset was multiplied by the number of living cells as determined by CASY Counter. If not mentioned otherwise, significance was determined using the Student's *t*-test and defined as follows: ^*^P < 0.05, ^**^P < 0.01 and ^***^P < 0.001. Bar graphs show mean ± s.e.m. for the group sizes as indicated in the figure legends.

## Results

### Ppef2 Expression Is Restricted to CD8^+^ cDC1

**Figure 1:**
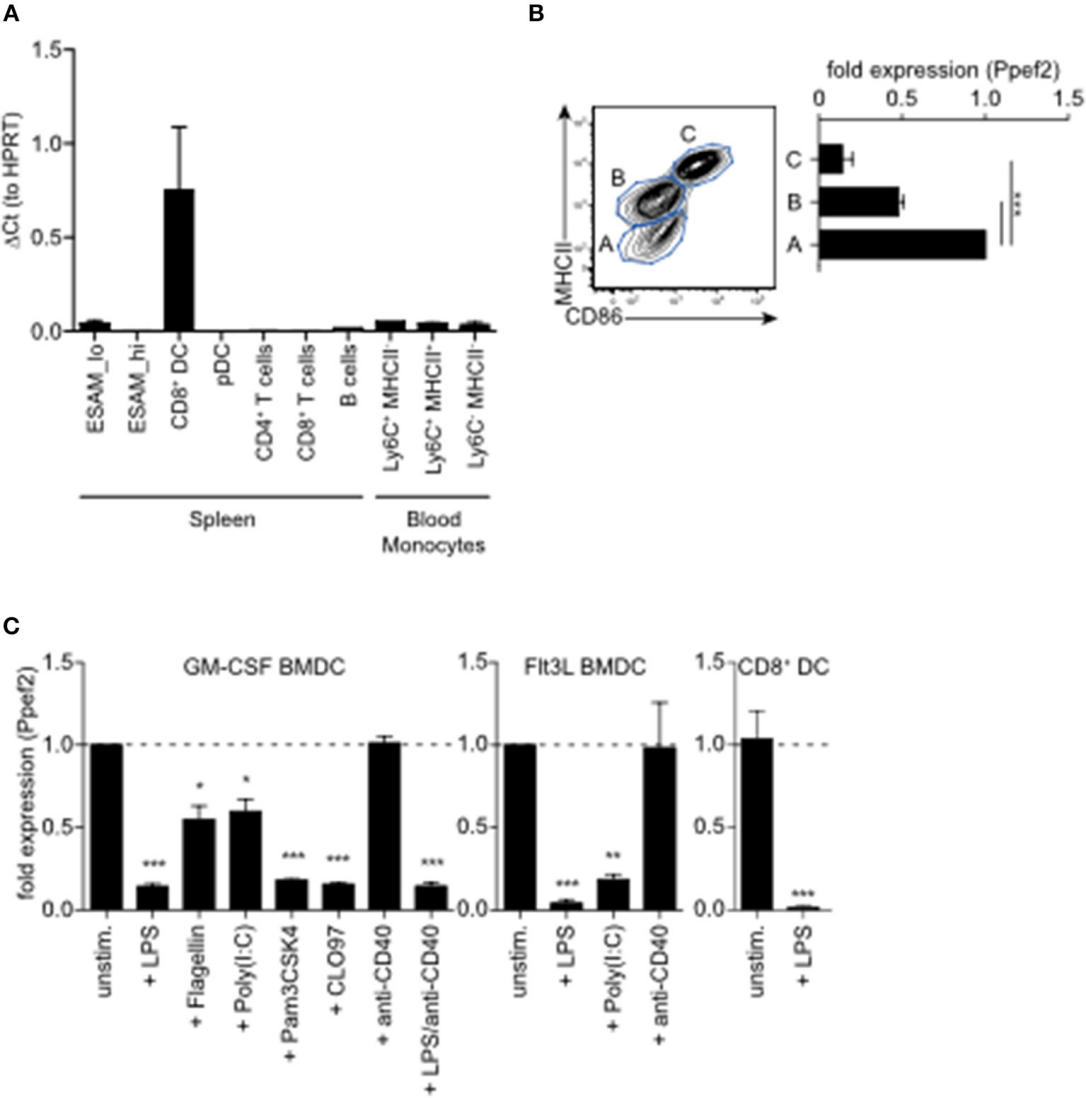
Ppef2 is predominantly expressed by CD8^+^ cDC1. **(A)** Gene expression profiling of spleen and blood cells after sorting. Shown is the quantitative real-time PCR result for Ppef2 of three independent sort experiments (*n* = 3 pooled mice per sort) ± SEM analyzed by the ΔCt method with HPRT as housekeeping gene. Cells were identified and sorted as ESAM^hi^ (CD11c^+^MHCII^+^CD11b^+^ESAM^hi^); ESAM^lo^ (CD11c^+^MHCII^+^CD11b^+^ESAM^lo^); CD8^+^DC (CD11c^+^MHCII^+^CD11b^−^CD8^+^); pDC (CD11b^−^SiglecH^+^); CD4^+^ T cells (CD3^+^CD4^+^); CD8^+^ T cells (CD3^+^CD8^+^); B cells (CD19^+^B220^+^); blood monocytes NK1.1^−^B200^−^CD115^+^CD11b^+^Ly6G^−^ cells with differential Ly6C and MHCII expression as indicated. Gating strategies are shown in Supplemental Figure 1B. **(B)** Ppef2 expression in GM-CSF cultured BMDCs of C57BL/6 mice after cell sorting on day 7 of culture based on the expression of CD11c, MHCII, and CD86. Error bars represent SEM of 3 independent experiments. **(C)** Ppef2 expression 16h after *in vitro* stimulation with the indicated TLR-ligands of GM-CSF- or Flt3L-cultured BMDCs of C57BL/6 mice, as well as spleen CD8^+^ DCs 16h after intravenous injection of 10 μg LPS as determined by qPCR. Bar graphs with SEM represent pooled data from independently performed cell cultures [GM-CSF BMDCs: unstim., LPS (*n* = 5); Flagellin, Poly(I:C), Pam3CSK4, CLO97, (*n* = 4); anti-CD40, LPS+anti-CD40 (*n* = 3); Flt3L BMDCs (*n* = 3); sorted CD8^+^ DCs (*n* = 4)]. Statistical analysis was performed using Student's *t*-test, with ^*^p < 0.05; ^**^p < 0.01; ^***^p < 0.001.

To analyse Ppef2-expression and regulation in greater detail, we purified lymphocyte populations from the spleen and blood of mice for gene expression analyses. Ppef2-expression was restricted nearly exclusively to MHCII^+^CD11c^+^CD8^+^ cDC1 in the spleen (Figure 1A), confirming earlier results (25). Other cells such as MHCII^+^CD11c^+^CD11b^+^ Esam^lo^ DCs (35); Supplementary Figure 1A), B cells and various distinct blood monocyte populations expressed only very low levels of Ppef2 mRNA (Figure 1A). Ppef2-expression was not detectable in pDCs, CD4^+^ T cells and CD8^+^ T cells (Figure 1A). Taken together, our analyses confirmed a highly specific expression of Ppef2 in CD8^+^ cDC1 as suggested by *Immgen* expression data collection (36) and our own previous findings (25). Also GMCSF-cultured BMDCs expressed Ppef2 (Figure 1B). Here, CD11c^+^ DCs of different activation stages as characterized by CD86 and MHCII surface expression, showed an inverted relation between Ppef2-mRNA expression and DC-activation (Figure 1B). In these cultures, MHCII^lo^CD86^lo^ immature DCs expressed the highest amount, while MHCII^int^CD86^lo^ semi-mature DCs expressed lower levels and most mature MHCII^hi^CD86^hi^ DCs expressed lowest levels of Ppef2 mRNA (Figure 1B). This Ppef2-expression pattern in spontaneously activated cultured BMDCs suggested that Ppef2 is down-regulated with increasing DC activation. Also, GM-CSF- or Flt3L-cultured BMDCs, as well as CD8^+^ cDC1 from spleens, stimulated with various toll-like receptor (TLR) ligands, such as LPS (TLR4), flagellin (TLR5), Poly(I:C) (TLR3), Pam3CSK4 (TLR1/2) or CLO97(TLR7/8), all downregulated Ppef2 mRNA (Figure 1C). In marked contrast, BMDC-activation by crosslinking of surface CD40 with anti-CD40 mAb alone (Figure 1C) did not cause down-regulation of Ppef2-expression (Figure 1C). Addition of LPS to anti-CD40 stimulated BMDCs caused down-regulation of Ppef2-mRNA, indicating a dominant effect of TLR- over CD40-signaling (Figure 1C). Taken together, our data indicates that CD8^+^ cDC1 selectively express Ppef2 mRNA, which is down-regulated rapidly upon DC-activation by TLR-signaling, but not by CD40-crosslinking.

### Generation of Ppef2-Deficient Mice

**Figure 2:**
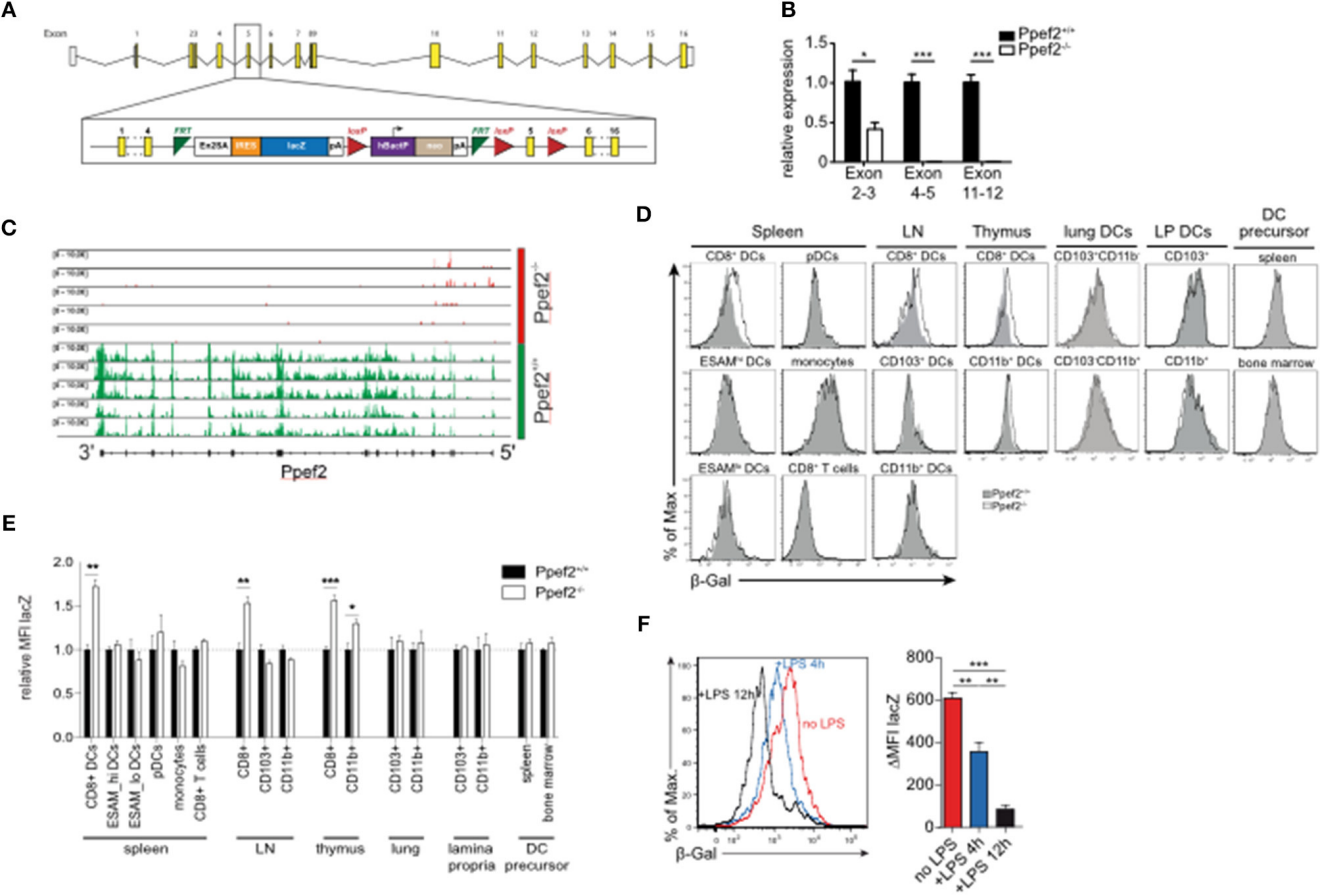
Ppef2-knockout strategy and LacZ-Reporter. **(A)** The Ppef2 locus with the knockout-first construct containing the gene trap between exon 4 and 5, as well as the floxed exon 5. **(B)** Ppef2 expression analysis of CD11c enriched splenocytes in Ppef2^+/+^ and *Ppef2*^−/−^ mice by quantitative real-time PCR. Three sets of intron-spanning primer pairs were used to amplify fragments from exons 2 to 3, 4 to 5, and 11 to 12. The ΔCt method was used to calculate the fold expression compared to Ppef2^+/+^ control samples. Data was normalized to HPRT (*n* = 3 mice). **(C)** Genomic distribution of reads across the Ppef2 gene locus in Ppef2^+/+^ and *Ppef2*^−/−^ CD8^+^ DCs. **(D)** Measurement of ß-Gal activity by flow cytometry in different cell types of the spleen, lymph node, thymus and bone marrow. Shown are representative FACS-plots of three experiments with similar outcome. **(E)** The mean fluorescence intensity (MFI) was calculated from one representative experiment out of two with identical outcome (*n* = 4). **(F)** Flow cytometric measurement of ß-Gal activity in splenic CD8^+^ DCs. Ppef2^lacZ/lacZ^ reporter mice (red, uninjected) were injected intravenously with LPS either 4 h (blue) or 12 h (black) before analysis. Shown are FACS-plots and statistics, where the ß-Gal signal of reporter mice was subtracted from the ß-Gal background signal of control mice (*n* = 18 mice). Statistical analyses were performed by using Student's *t*-test, with ^*^p < 0.05; ^**^p < 0.01; ^***^p < 0.001.

To explore the role of Ppef2 *in vivo*, we next generated Ppef2-deficient (*Ppef2*^−/−^) mice using the knock-out first strategy (37) by introducing a gene-trap between exons 4 and 5 as well as flanking loxP-sites of exon 5 (Figure 2A). DCs from *Ppef2*^−/−^ animals lacked Ppef-mRNA, as detected by quantitative PCR (Figure 2B) as well as the analysis of reads from mRNA-sequencing of CD8^+^ cDC1 (Figure 2C). Similar results were obtained from total splenocytes, where also no Ppef2-mRNA was detectable (data not shown). These analyses revealed complete lack of Ppef2-transcripts beyond exon 3, indicating successful knockdown of gene expression (Figures 2B,C). Due to lack of functioning Ppef2-specific antibodies, further direct studies of Ppef2-protein expression were not possible. However, the introduction of a gene trap/lacZ construct allowed indirect monitoring of expression and regulation of Ppef2 using ß-galactosidase (ß-Gal) as a surrogate. FACS-analysis of various cell types from spleen, lymph nodes and thymus confirmed the highly selective expression of Ppef2/ß-Gal in CD8^+^ DCs of spleen and lymph nodes as well as CD8^+^ cDC1 and CD11b^+^ cDC2 of thymus (Figures 2D,E). Other cells such as CD8^+^ T cells, LN CD103^+^ cDC1 and CD11b^+^ cDC2, spleen CD11b^+^ESAM^hi^ and CD11b^+^ESAM^lo^ cDC2, monocytes (Figures 2D,E), CD4^+^ T cells, B cells and neutrophils (data not shown), did not express Ppef2/ß-Gal *in vivo*. Furthermore, lineage^−^MHCII^−^CD11c^low^CD43^+^sirp-α^+^ DC-precursors (Supplementary Figure 1E) (38), which give rise to both, CD8^+^ and CD103^+^ cDC1 in lymphoid and non-lymphoid tissues (1), did not express Ppef2/ß-Gal (Figures 2D,E). Interestingly, while CD8^+^ cDC1 were Ppef2/ß-Gal-positive, CD103^+^ cDC1 in lung and intestinal lamina propria tissue, which develop from the same pre-DC were Ppef2/ß-Gal-negative (Figures 2D,E). This indicates that Ppef2/ß-Gal-expression is regulated after the pre-DC stage and might be sensitive to signals from tissue milieu or the environment. CD8^+^ cDC1 also showed strong down-regulation of Ppef2/ß-Gal upon stimulation with TLR4-ligand LPS *in vivo* (Figure 2F), confirming the shut-down of Ppef2 expression upon activation of DCs *in vivo*. These data demonstrate the successful knockout of Ppef2, its specific expression in the CD8^+^ cDC1 subset and Ppef2 down-regulation upon DC-activation.

### Ppef2-Deficient Mice Have Normal Numbers of DCs

**Figure 3:**
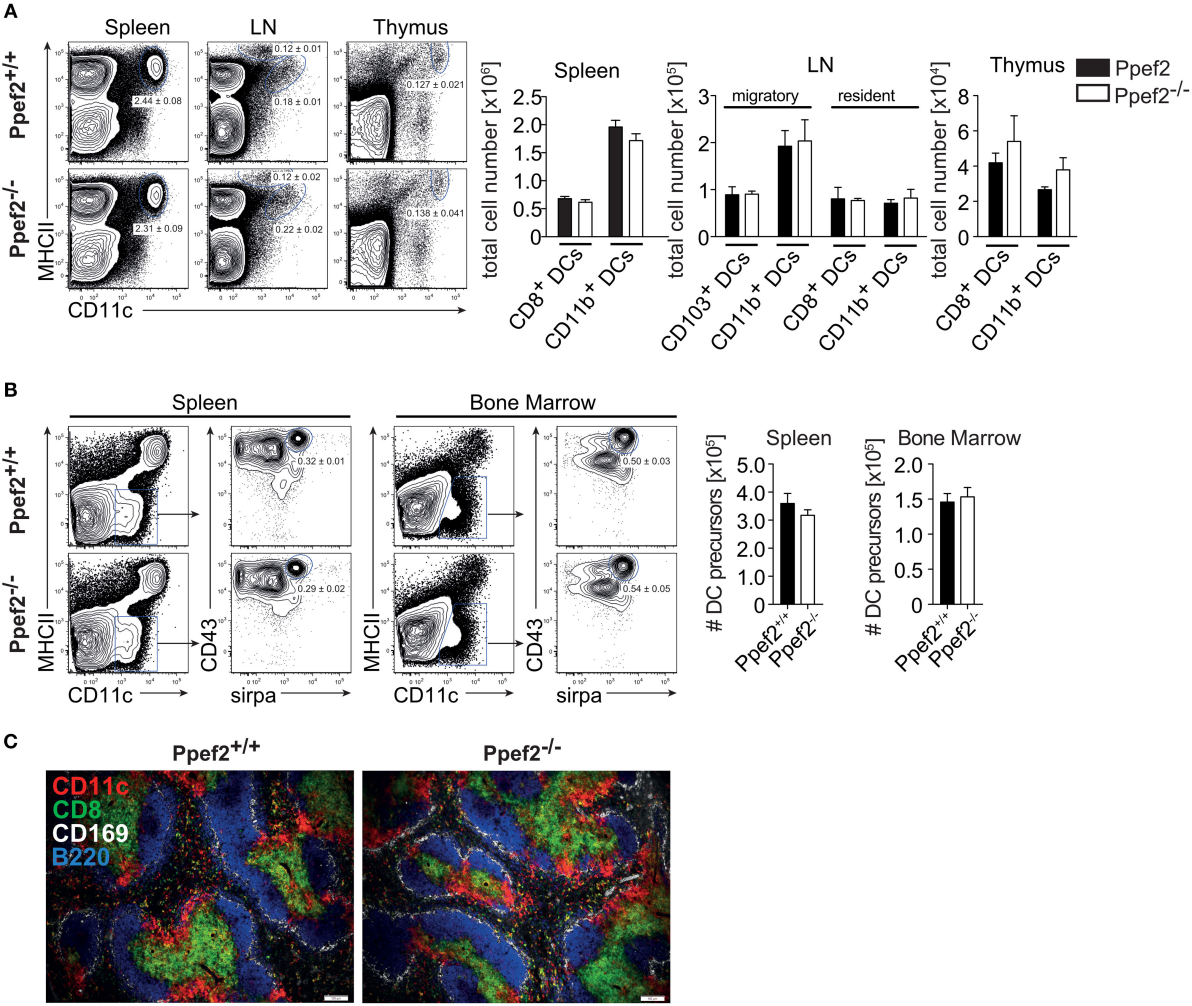
*Ppef2*^−/−^ -mice have normal frequencies of DCs and DC-precursors. **(A)** DCs of spleen, LN and thymus were stained with CD11c and MHCII. Shown are representative FACS plots with the average DC percentages ± SEM of pooled data (spleen, 8 experiments (*n* = 36); sLN, 4 experiments (*n* = 23); thymus, 2 experiments (*n* = 6) as well as the corresponding total cell numbers. **(B)** The frequency of DC precursors from spleen (*n* = 3) and bone marrow (*n* = 4). Shown is one representative experiment out of two with similar outcome. Statistical analysis of **(A, B)** was performed using Student's *t*-test and all *p*-values were above 0.05. **(C)** Acetone fixed spleen sections of Ppef2^+/+^ and *Ppef2*^−/−^ mice were stained with antibodies against CD11c (red), CD8 (green), CD169 (white), and B220 (blue). Scale bars represent 100 μm.

We next analyzed if lack of Ppef2 would have an impact on the composition of the DC-compartment or other cells *in viv*o. However, we could not detect differences as compared to Ppef2^+/+^ mice, when we analyzed frequencies and cell numbers of DC subsets in spleens, lymph nodes or thymi of *Ppef2*^−/−^ mice (Figure 3A). Also, analysis of DC subsets in the lung, intestinal lamina propria, liver and skin dermis could not reveal statistically significant differences of *Ppef2*^−/−^ DCs subsets *in vivo* (Supplementary Figure 2). Further analysis of other hematopoietic cell subsets (Supplementary Figure 3) with additional markers (Supplementary Table 1) or MHCII^−^CD11c^−^CD43^+^sirpa^+^ DC-precursors in bone marrow and spleen (Figure 3B) also showed no differences between *Ppef2*^−/−^ and wild-type controls. The spleen architecture of *Ppef2*^−/−^ animals and the positioning of DCs was normal as revealed by histological immunofluorescence analyses (Figure 3C). Taken together, lack of Ppef2 did neither alter the composition of DC subsets nor the haematopoietic compartment in general.

### Lack of Ppef2 Causes an Increased Rate of Apoptosis and Decreased Survival Rate in DCs

**Figure 4:**
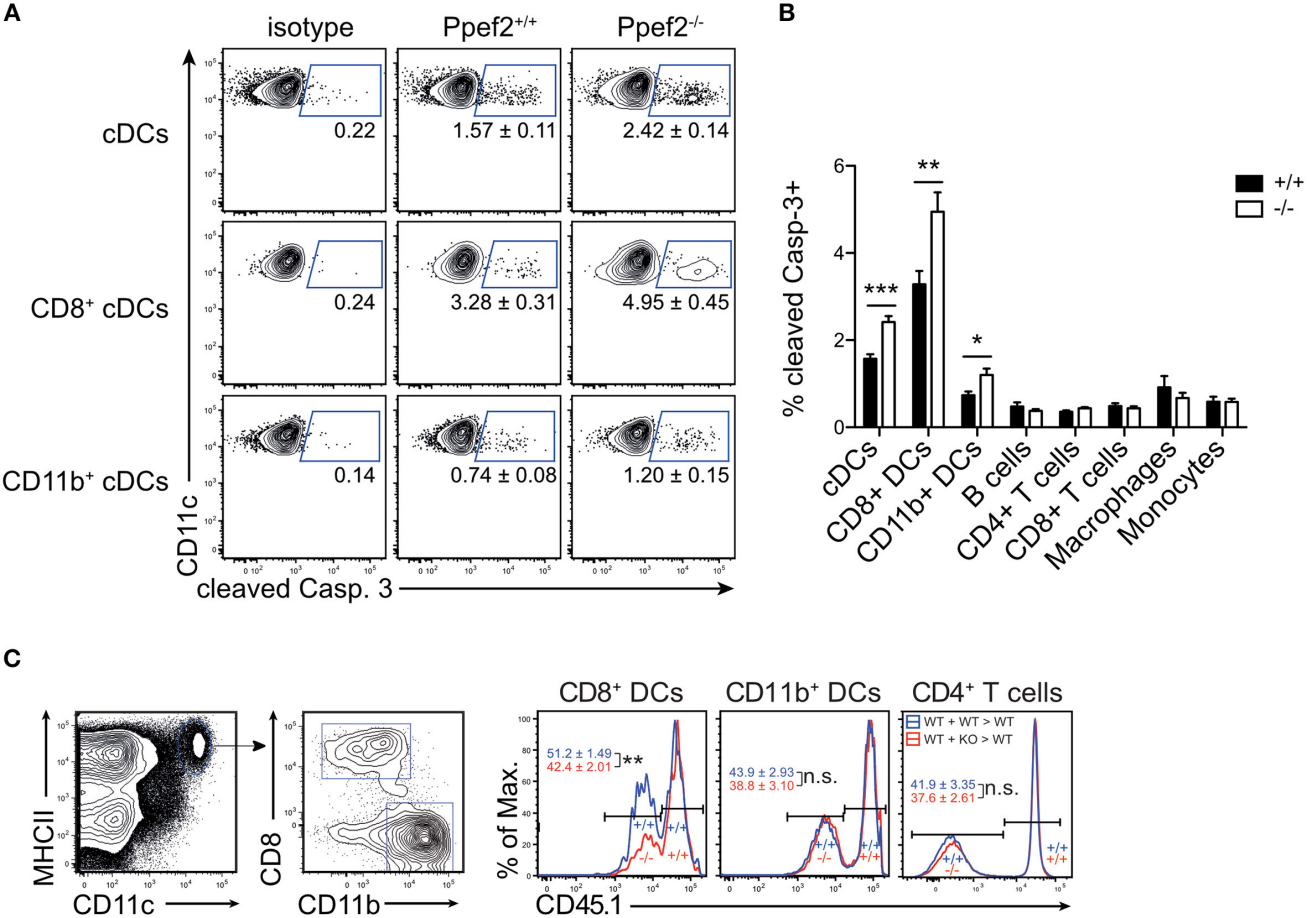
Ppef2-deficiency causes increased levels of cleaved caspase-3 in DCs. **(A)** Splenocytes were stained intracellularly for cleaved caspase-3 and analyzed by flow cytometry. Shown are representative FACS-plots of two experiments with similar outcome (*n* = 4 each). The corresponding statistics of the pooled data (*n* = 8) are shown in **(B)** together with the statistical analysis of other cell types. Statistical analysis was performed using Student's *t*-test, with ^*^p < 0.05; ^**^p < 0.01; ^***^p < 0.001. **(C)** Mixed bone marrow chimeras were produced by irradiation of CD45.1^+^ recipients and reconstitution with a 1:1 mix of Ppef2^+/+^ (CD45.1^+^) and Ppef2^+/+^ (CD45.2^+^) bone marrow (+/+: +/+ > +/+), or a 1:1 mix of Ppef2^+/+^ (CD45.1^+^) and *Ppef2*^−/−^ (CD45.2^+^) bone marrow (+/+: –/– > +/+). Mixed bone marrow chimeras were analyzed 8–10 weeks after reconstitution by gating on CD11c^+^MHCII^+^CD8^+^CD11b^−^ cDC1, CD11c^+^MHCII^+^CD8^−^CD11b^+^ cDC2, CD4^+^TCRβ^+^ T cells and CD45.1. Shown are representative FACS-plots of three independently performed experiments with similar outcome (*n* = 11) and statistical analysis was performed using Student's *t*-test, with ^**^p < 0.01.

As Ppef2 has been linked functionally to cell survival and apoptosis in previous *in vitro* studies in human cells (32), we next tested if *Ppef2*^−/−^ DCs would show increased apoptosis. We analyzed CD11c^+^MHCII^+^ DCs from spleens for presence of cleaved caspase 3 and detected significantly more Casp3^+^CD11c^+^MHCII^+^ DCs in *Ppef2*^−/−^ mice as compared to wild type controls (Figures 4A,B). More detailed analysis showed that *Ppef2*^−/−^ CD8^+^ cDC1 contained the highest frequencies of Casp3^+^ apoptotic cells, but also CD11b^+^ cDC2 were affected to some extent (Figures 4A,B). In contrast, other lymphocytes from *Ppef2*^−/−^ mice such as B cells, T cells, macrophages or monocytes did not show any elevated levels of Casp3 (Figure 4B). Taken together, these findings suggest that the absence of Ppef2 leads to increased rates of apoptosis in CD8^+^ cDC1. We next wondered if this would influence their competitive behavior *in vivo*. To test this, we chose a competitive situation and generated radiation bone marrow chimeras, which were reconstituted with a mix of bone marrow derived from CD45.1^+^ Ppef2^+/+^ and CD45.1^−^ *Ppef2*^−/−^ mice at a 1:1 ratio. The analysis of these chimeras showed that CD45.1^−^ *Ppef2*^−/−^ CD8^+^ cDC1 were non-competitive as compared to Ppef2^+/+^ CD8^+^ cDC1 in the same animal, as reconstitution was significantly less efficient for *Ppef2*^−/−^ CD8^+^ cDC1 as compared to Ppef2^+/+^ CD8^+^ cDC1 (Figure 4C). This effect was specific for CD8^+^ cDC1, as *Ppef2*^−/−^ CD11b^+^ cDC2 and CD4+ T cells developed normally and generated frequencies comparable to those of Ppef2^+/+^ CD11b^+^ cDC2 (Figure 4C). Therefore, although under non-competitive conditions *Ppef2*^−/−^ DCs developed in normal numbers (Figure 3), they were outcompeted by Ppef2^+/+^ DCs during reconstitution upon lethal irradiation.

### Transcriptome Analysis of *Ppef2^−/−^* CD8^+^ cDC1 Reveals Regulation of Apoptosis and Cell Survival Genes

**Figure 5:**
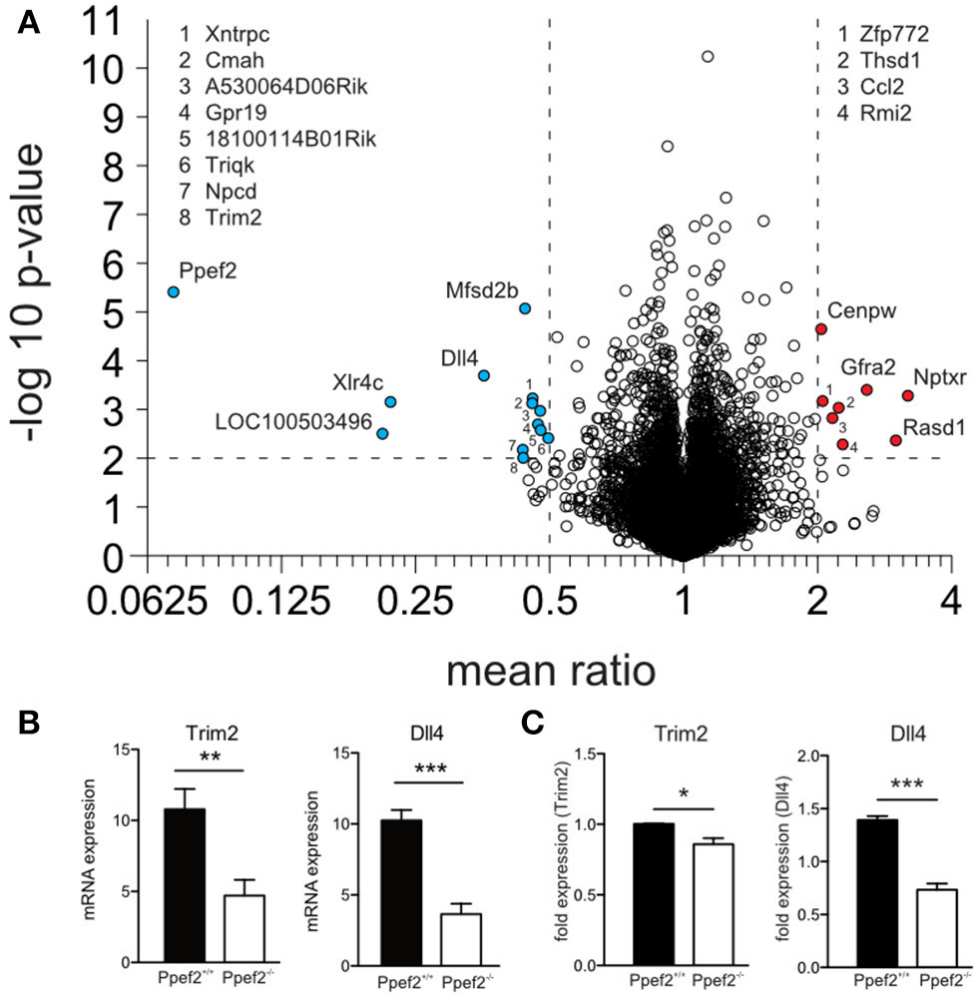
RNA-sequencing reveals changes in RNA-expression of *Ppef2*^−/−^ CD8^+^ cDC1. **(A)** CD8^+^ DCs were purified by flow cytometry from cell suspensions of 3 pooled spleens as live MHCII^+^CD11c^+^CD11b^−^CD8^+^ cells to purity of >95%. 15 spleens from Ppef2^+/+^ or *Ppef2*^−/−^ mice were used to generate 5 samples each for RNA-sequencing. Shown is the volcano plot analysis of sorted CD8^+^ DCs. Fold change of−2 (a, blue) and +2 (a, red), and a *p*-value ≤ 0.01 were chosen as cut-off. Ppef2, protein phosphatase EF-hands 2; LOC100503496, uncharacterized transcript LOC100503496; Xlr4c, X-linked lymphocyte-regulated 4C; Dll4, delta-like ligand 4; Trim2, tripartite motif-containing 2; Npcd, neuronal pentraxin chromo domain; Mfsd2b, major facilitator superfamily domain containing 2B; Cmah, cytidine monophospho-N-acetylneuraminic acid hydroxylase; Xntrpc, Xndc1-transient receptor potential cation channel, subfamily C, member 2; A530064D06Rik, Riken cDNA A530064D06 gene; 1810014B01Rik, Riten cDNA 1810014B01 gene; Triqk, triple QxxK/R motif containing; Nptxr, neuronal pentraxin receptor; Rasd1, RAS, dexamethasone-induced 1; Gfra2, glial cell line derived neurotrophic factor family receptor alpha 2; Rmi2, RMI2, RecQ mediated genome instability 2; Thsd1, thrombospondin, type I, domain 1; Ccl2, chemokine (C-C motif) ligand 2; Zfp772, zinc finger protein 772; Cenpw, centromere protein W. **(B)** Boxplots represent normalized expression with 0,1 quantile, 0.9 quantile and all single points (each group *n* = 5) ^**^p < 0.01, ^***^p < 0.001 for Trim2 and Dll4 in Ppef2^+/+^ and *Ppef2*^−/−^ cells. **(C)** qPCR of Trim2 and Dll4 in sorted spleen CD8^+^ DCs. Spleen DCs were sorted as CD11c^+^MHCII^+^CD8^+^CD11b^−^ in three independent experiments and three Ppef2^+/+^ or *Ppef2*^−/−^ mice were pooled for every sort. Statistical analysis was performed using Student's *t*-test, with ^*^p < 0.05; ^***^p < 0.001.

To analyse if absence of Ppef2 might alter gene expression which eventually controls DC survival, we performed mRNA sequencing of sorted splenic CD8^+^ cDC1 (Figure 5A). A total of 10,766 genes were detected and the read counts for Ppef2 were used as internal control. *Ppef2*^−/−^ DCs did not show any Ppef2-reads above background (Figure 5A), confirming the efficiency of the Ppef2-knockout. Analysis of differentially expressed genes revealed 13 down- (Figure 5A, blue) and 8 up-regulated genes (Figure 5A, red) that were at least 2-fold different with a *p*-value ≤ 0.01. Among the differentially regulated genes, *tripartite motif-containing 2* (Trim2) was downregulated more than 2-fold (Figure 5B); this gene is of particular interest because it binds to the phosphorylated form of the pro-apoptotic Bim, mediating its ubiquitination for degradation (39). Delta-like ligand 4 (Dll4), a ligand for Notch (40), was down-regulated 2.8-fold in Ppef^−/−^ CD8^+^ cDC1 (Figure 5B). Notch signaling is known to be crucial for lymphocyte development and function (41) and signaling via Notch maintains CD11b^+^ cDC2 (42). To validate the mRNA sequencing results, we performed a qPCR of Trim2 and Dll4 on sorted CD8^+^ cDC1 of the spleen (Figure 5C). Both mRNAs were down-regulated significantly in *Ppef2*^−/−^ CD8^+^ cDC1 (Figure 5C), confirming the results from mRNA-sequencing (Figures 5A,B).

### *Ppef2^−/−^* DCs Have Elevated Levels of Cytoplasmic Pro-Apoptotic Bim

**Figure 6:**
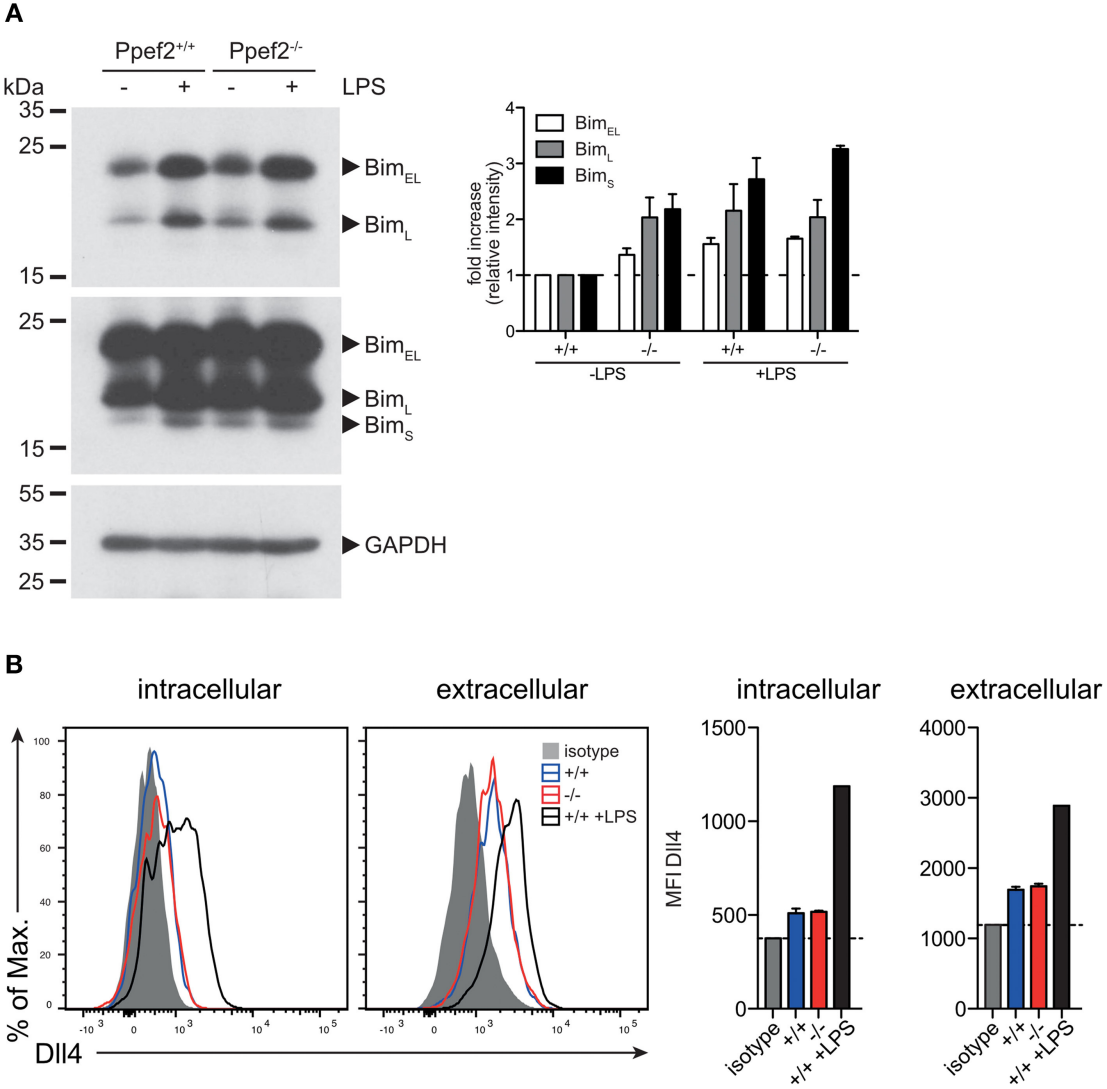
*Ppef2*^−/−^ DCs have elevated levels of pro-apoptotic BIM. **(A)** Western blot for Bim was performed with 30 μg cell lysates of GM-CSF BMDCs either unstimulated or LPS matured. Exposure for 30 s allowed detection of BimEL and BimL isoforms (**A** left, top panel); 5 min exposure revealed the BimS isoform in addition (**A** left, middle panel). Intensities of the bands for all three Bim isoforms were quantified relative to the GAPDH loading control (**A** left, lower panel) by using ImageJ and fold increase was calculated relative to the untreated Ppef2^+/+^ control (**A**, right hand panel). Two western blots were performed and shown are pooled normalized intensities. **(B)** Surface staining of Dll4 was performed with fluorescently labeled antibody and protein abundance was measured by flow cytometry. Shown are representative FACS plots (left) and mean ± SEM of the mean fluorescence intensity of *n* = 3 mice.

We next analyzed protein levels of Trim2 by Western blot analyses but could not detect significantly different expression between *Ppef2*^−/−^ and Ppef2^+/+^ BMDC (data not shown). Next we performed western blot analyses of Bim in GM-CSF cultured BMDCs (Figure 6A). As Bim increases after stimulation with TLR ligands (14), we stimulated BMDCs with LPS as positive control. All three Bim isoforms, Bim_EL_, Bim_L_, and Bim_S_ can promote apoptosis (43) and could be readily detected (Figures 6A,B). Quantification of Bim relative to the GAPDH loading controls showed that in unstimulated *Ppef2*^−/−^ BMDCs more Bim of all isoforms was present as compared to controls (Figure 6A). Bim levels in unstimulated *Ppef2*^−/−^ BMDCs were similar to the increased levels found in LPS-induced Ppef2^+/+^ BMDCs (Figure 6A). Stimulation of *Ppef2*^−/−^ DCs with LPS could increase Bim only marginally, while it increased in Ppef2^+/+^ wt DCs as described previously (14) (Figure 6A).

This suggests that Bim-mediated apoptosis might cause the alterations observed in *Ppef2*^−/−^ DCs and cause their competitive disadvantage (Figure 4C).

Although expression of Dll4 is generally low in cDCs (36), we saw changes of Dll4-mRNA-expression in CD8^+^ cDC1 of *Ppef2*^−/−^ mice (Figures 5A,B). Therefore, we next analyzed Dll4-protein levels. Spleen cDCs showed only minor intracellular staining over background (isotype) control, with slightly elevated surface expression levels (Figure 6B). As published previously, LPS-treatment augmented DII4-expression significantly (44). However, Ppef2^+/+^ and *Ppef2*^−/−^ DCs showed comparable Dll4 levels, indicating that the difference in Dll4-mRNA-expression could not be confirmed on surface protein levels. Taken together, Ppef2-deficiency seems to cause increased levels of pro-apoptotic Bim in CD8^+^ cDC1.

### *Ppef2^−/−^* DCs Display Decreased Capacity of CD8^+^ T Cell Priming

**Figure 7:**
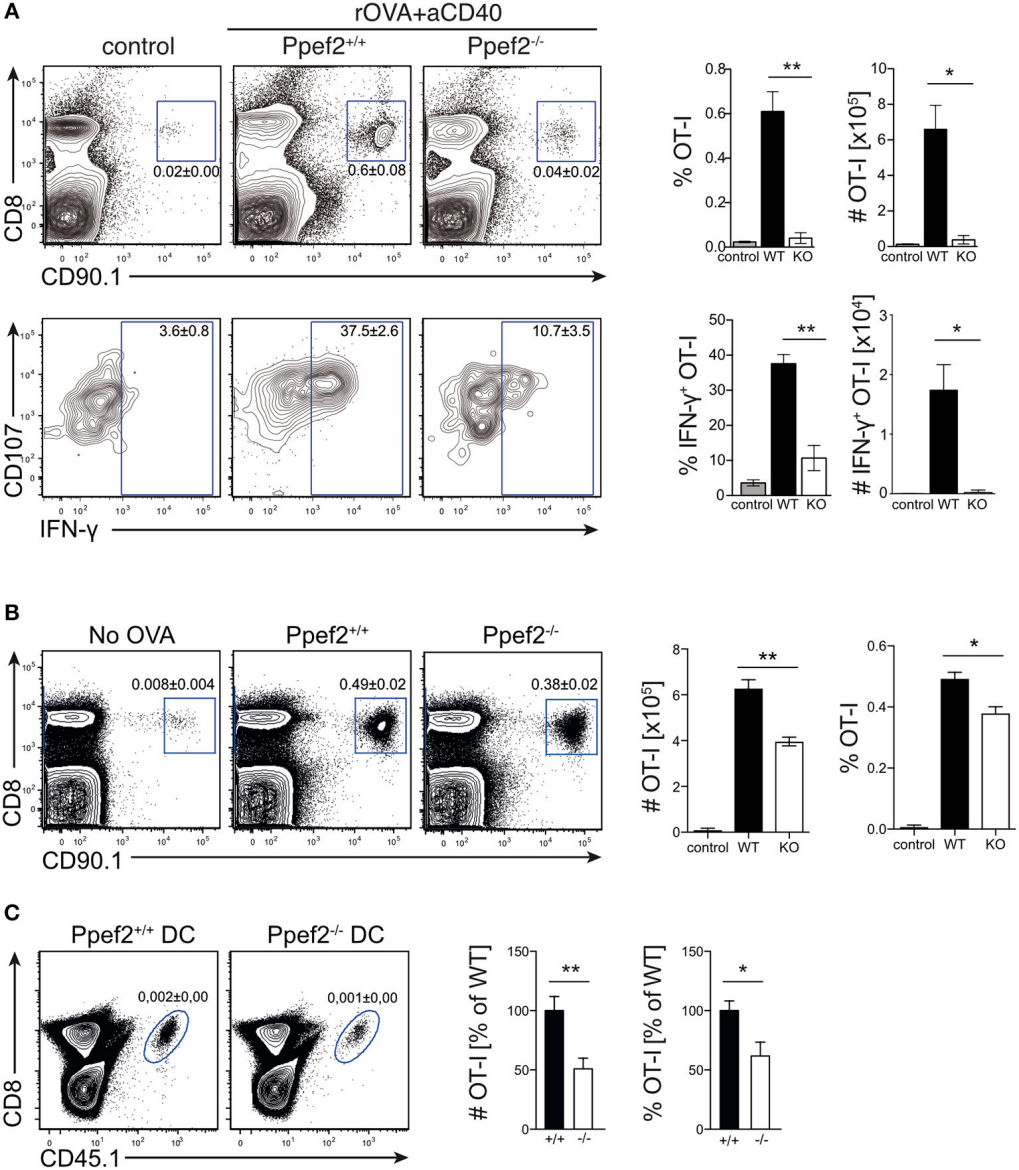
*Ppef2*^−/−^ DCs display decreased CD8^+^ T cell priming capacities. **(A)** 5 × 10^5^ congenitally marked CD90.1^+^ OT-I T cells were adoptively transferred either in Ppef2^+/+^ or *Ppef2*^−/−^ mice, which received 30 μg OVA protein and 100 μg CD40-specific mAb intravenously 1 day later or received OT-I T cells only (control). On day 7 post immunization spleens were analyzed for frequencies and total cell numbers of CD90.1^+^CD8^+^ OT-I cells as shown in the respective bar graphs (upper panel). Spleen cells were cultured *in vitro* with OVA257 peptide in the presence of CD107a-specific mAb and subsequently stained for CD8 and IFN-γ (lower panel) to determine IFN-γ-producing OT-I T cell frequencies and total cell numbers (bar graphs). Shown are results from one experiment out of two with similar results and *n* = 4 mice per group. **(B)** 5 × 10^5^ congenitally marked (CD90.1) OT-I T cells were adoptively transferred either in Ppef2^+/+^ or *Ppef2*^−/−^ mice, which received 100 μg OVA protein intravenously 1 day later. Mice were sacrificed 3 days after OVA administration and spleens were analyzed by flow cytometry for OT-I T cell proliferation. Shown are representative FACS-plots of OT-I T cells of one out of two independently performed experiments with similar outcome. Data from two independent experiments were pooled (*n* = 6) to determine the total cell number and frequencies of CD90.1^+^CD8^+^ OT-I T cells. **(C)** 1.5 × 10^5^ Ppef2^+/+^ or *Ppef2*^−/−^ DCs were pulsed with OVA257 peptide and transferred i.v. into H-2K^bm1^ recipient mice. 24 h later H-2K^bm1^ OT-I T cells were transferred and mice were analyzed 3 days later for presence of CD45.1^+^CD8^+^ OT-I T cells by flow cytometry. Shown are representative FACS-plots of the gated CD45.1^+^CD8^+^ OT-I T cells. Two independently performed experiments were pooled (*n* = 6) to determine the relative frequency and total cell number of CD45.1^+^CD8^+^ OT-I T cells from the gates shown in **(C)**. Statistical analysis was performed using Student's *t*-test, with ^*^p < 0.05; ^**^p < 0.01.

The life-span of DCs directly influences antigen-specific expansion of T cells (13, 17). We therefore wondered whether Ppef2-deficiency would alter antigen-presentation and T cell priming *in vivo*. As infection models with pathogens or protein immunization with adjuvants contain TLR-signals which rapidly downregulate Ppef2 in Ppef2^+/+^ DCs (Figures 1C, 2F), such models render *Ppef2*^−/−^ and Ppef2^+/+^ mice quite similar with respect to low Ppef2-levels. CD40-crosslinking did not induce Ppef2-downregulation (Figure 1C). Therefore, we adoptively transferred TCR-transgenic CD90.1^+^ OT-I T cells into Ppef2^+/+^ or *Ppef2*^−/−^ mice and injected ovalbumin (OVA) as cognate antigen together with an agonistic anti-CD40 mAb for DC-activation (45). Here, cross-presentation by CD8^+^ cDC1 should be the main mechanism for CTL-priming. While OT-I cells expanded strongly in the spleens of Ppef2^+/+^ mice, only marginal OT-I expansion was detected in *Ppef2*^−/−^ mice, similar to control mice that received OT-I cells alone (Figure 7A, upper panel). The lack of efficient OT-I cross-priming in *Ppef2*^−/−^ mice was evident from the percentage of OT-I cells found on day 7 post priming in the spleens of mice, as well as from the total OT-I numbers (Figure 7A, upper panel). When OT-I cells were restimulated with the cognate OVA257 peptide *in vitro*, only very low frequencies and total numbers of CD107^+^IFN-γ^+^ OT-I effector T cells were found in spleens of *Ppef2*^−/−^ mice (Figure 7A, lower panel). In contrast, OT-I cells primed in Ppef2^+/+^ mice were readily producing IFN-γ^+^ (Figure 7A, lower panel). We next tested antigen presentation in absence of inflammatory stimuli and injected OVA in PBS. 3 days later *Ppef2*^−/−^ mice showed a significantly reduced OT-I T frequency and nearly 40% reduced OT-I T cell numbers (Figure 7B). This data suggests that cross-presentation of OVA-protein by immature DCs in absence of inflammatory stimuli as well as by CD40-matured DCs is severely reduced in *Ppef2*^−/−^ mice.

To exclude differential antigen uptake or processing of OVA antigen by *Ppef2*^−/−^ DCs *in vivo*, we next transferred OVA257-peptide pulsed *Ppef2*^−/−^ or Ppef2^+/+^ DCs into H-2K^bm1^ recipient mice. Here, direct peptide loading of DCs avoids the necessity for antigen-processing, while usage of H-2K^bm1^ mice as recipients does not allow peptide presentation by cells of the host recipient mice, as the H-2K^bm1^ mutation of the H2-K^b^ gene leads to a failure to present OVA257 on MHC class I (46). Adoptively transferred CD45.1^+^H-2K^bm1^ OT-I T cells showed strongly reduced frequencies and total cell numbers when stimulated by *Ppef2*^−/−^ DCs as compared to Ppef2^+/+^ DCs (Figure 7C). Taken together, our data suggests that Ppef2 expression in CD8^+^ cDC1 ultimately contributes to efficient cross-presentation of protein antigen.

## Discussion

Currently very little is known about Ppef2. In this study we show that among hematopoietic cells specifically CD8^+^ cDC1 express the phosphatase Ppef2, which is down-regulated upon DC-activation by TLR-ligands, but not by CD40-engagement. Down-regulation of Ppef2 increases apoptosis and limits antigen-presenting cell functions of CD8^+^ cDC1.

Transcriptional profiling showed that Ppef2 is expressed with high specificity in CD8^+^ cDC1 and confirmed the microarray data available from the Immgen consortium (36). Although CD11b^+^ cDC2 and monocytes were also reported to express very low levels of Ppef2 mRNA, this did not translate into protein as determined by β-galactosidase, which is included in the gene-trap cassette of *Ppef2*^−/−^ mice. However, tissue CD103^+^ cDC1 show some Ppef2-mRNA expression according to the Immgen database (36), but do not express significant amounts of Ppef2 (ß-Gal)-protein. CD11b^+^ cDC2 of the thymus also express the Ppef2-reporter. According to the Immgen-database, both thymic DC-subsets, CD8^+^ DCs and CD8^−^ DCs express high levels of Ppef2-mRNA, which is different from the spleen,where CD8^+^ cDC1, but not CD11b^+^ cDC2 express Ppef2-mRNA (36). As Ppef2 shares promoter organization with CD11c and the respective promoter motive is sufficient to drive DC-specific gene expression in all CD11c^+^ DCs (25), the cDC1-specific Ppef2-expression is surprising. This suggests that Ppef2-expression is most likely controlled on additional levels leading to differential expression in distinct DC subpopulations. More specifically, CD8^+^ and CD103^+^ cDC1 subsets differentiate from the same Ppef2-negative pre-DC precursor into different environments. While CD8^+^ cDC1 are mostly resident in lymphoid organs, CD103^+^ cDC1 develop in barrier organs such as skin and mucosal tissues. Here, CD103^+^ DCs might routinely encounter TLR-signals from skin, lung and intestinal microbes, which could eventually cause down-regulation of Ppef2 in the steady-state. In contrast, CD8^+^ cDC1 in lymph nodes and spleen should not readily receive TLR-signals due to their localization and might therefore continue to express Ppef2.

In the case of an infection, exposure of cDC1 to TLR-ligands also down-regulates Ppef2-expression in mature DCs. This is in contrast to CD40-signals; although both signaling pathways utilize TNF-associated factor (TRAF) 6, CD40 and TLR-signaling leading to NF-κB activation vary from each other (47). CD40 mediated TRAF-signaling involves also TRAF2 and 3 (48), while TLR-signaling involves IL-1R-associated kinases (IRAKs). In addition, CD40 signaling can result in protein kinase B (Akt) activation (49), which may promote survival, while TLR signaling was shown to induce JUN N-terminal kinase (JNK), which may cause apoptosis (50). This data fits to the hypothesis that CD40 induces pro-survival signaling, whereas TLR signaling is rather pro-apoptotic and involves mitogen-activated protein kinases, which regulate Ppef2 expression and possibly promote apoptosis. In contrast, pro-survival TRAF6-CD40 signaling might utilize Akt not altering the expressing of Ppef2. We observed down-regulation of Ppef2 expression also after spontaneous maturation of DCs in culture and other studies have shown similarities between LPS activated and spontaneously activated cells *in vitro*, when analyzing microarray data (51). The majority of changes in gene expression during DC-activation are reductions of expression (52). Therefore, it is difficult to conclude whether loss of expression of Ppef2 in mature DCs is simply a consequence of co-downregulation of other genes, which may be dispensable for mature DCs, or whether it is a specific regulation in order to limit the lifespan of DCs.

The only functional Ppef2-study published so far investigated the anti-apoptotic role of Ppef2 in oxidative stress by suppression of Apoptosis signal-regulating kinase 1 (Ask1) in immortalized cell lines (32). Ppef2 caused potent dephosphorylation of Ask1 at the threonine residue 838, suppressing Ask1 activity (53). When Ask1 and Ppef2 were co-expressed *in vitro*, suppression of Ask1 by Ppef2 led to a decrease in caspase-3 activation (32). It was concluded that the phosphatase Ppef2 functions to control Ask1 activity to prevent apoptosis under oxidative stress conditions. Ppef2 is also up-regulated in mouse hippocampus after hypoxia-ischemia, along with several other transcripts associated with neuroprotection (54). However, western blot analyses of *Ppef2*^−/−^ DCs revealed no changes in Ask1 expression or phosphorylation (data not shown). Instead, mRNA-sequencing revealed Trim2 and Dll4 being expressed at reduced levels in *Ppef2*^−/−^ CD8^+^ cDC1.

Trim2, an E3 Ligase, ubiquitinates pro-apoptotic Bim for proteasomal degradation (39). *Ppef2*^−/−^ DCs showed an increase in Bim, for which a decrease in Trim2 could be responsible, although we were unable to confirm alterations of Trim2 protein levels in *Ppef2*^−/−^ DCs (data not shown). In conclusion, we showed that loss of Ppef2 led to a decrease of Trim2 mRNA which can influence the abundance of Bim. Being a regulator of DC apoptosis, Bim would therefore be responsible for the increase in apoptotic active caspase-3^+^ DCs observed in *Ppef2*^−/−^ mice.

Interestingly, we also observed increased caspase-3 levels in CD11b^+^ cDC2 of *Ppef2*^−/−^ mice *in vivo*, although these DCs do not express Ppef2. Eventually indirect secondary effects or the uptake of apoptotic cleaved caspase-3^+^CD8^+^ cDC1 by CD11b^+^ cDC2 might account for this finding. Recently, it has been shown that oxysterol-metabolizing enzyme expressing CD8^+^ (XCR1^+^) cDC1 do influence homeostasis and positioning of CD11b^+^ (DCIR2^+^) cDC2 (55), indicating a possible cross-talk between both DC-subsets. However, the exclusivity of the competitive disadvantage observed for CD8^+^, but not CD11b^+^ DCs, during bone marrow chimera reconstitution suggests that Ppef2-deficiency directly affects CD8^+^ cDC1 only.

Although the rate of apoptosis was increased in CD8^+^ cDC1 from *Ppef2*^−/−^ mice, we did not find diminished total numbers or frequencies of CD8^+^ cDC1. Also short-term or pulse-chase BrdU-labeling experiments could not reveal statistically significant differences for DC proliferation or turnover, which might compensate for increased rates of DC-death (data not shown). As only 5% of all DCs proliferate in steady-state (12), BrdU-incorporation might not be sensitive enough to reveal subtle changes in compensatory proliferative rates. Also serum levels of Flt3L were not elevated, as reported in mice which lack DCs due to expression of diphteria toxin (56). Eventually, alterations in precursor-product relations might account for compensatory effects. However, we did not detect elevated numbers of pre-DCs, nor did they show an increased proliferative rate (data not shown).

Increased apoptosis of DCs should lead to decreased T cell priming capacity. In our experiments we avoided to activate DCs with TLR-ligands, as fully matured DCs naturally down regulate Ppef2 and as such would not be different from *Ppef2*^−/−^ DCs. T cell proliferation was substantially reduced upon priming by *Ppef2*^−/−^ DCs. Also cross-presentation of OVA-protein by CD40-matured CD8^+^ cDC1 was strongly reduced in *Ppef2*^−/−^ mice, indicating that DCs have limited T cell priming ability when they lack Ppef2. This might hint for a DC-intrinsic mechanism, which could limit antigen-specific T cell responses for protection from excessive immune activation to avoid collateral tissue damage and autoimmunity, which has been observed with apoptosis-resistant DCs (13–17).

Although its sequence is highly conserved in humans, hPpef2 does not show a similar DC-specific expression pattern (57), but is rather restricted to the retina. Yet, Ppef2 was identified as a survival phosphatase also in human HELA-cells in an RNA interference screening (58). Eventually, other members of the PPP phosphatase family such as PPP3CC, which is expressed in human DCs (59), might play corresponding roles.

Taken together, the Ppef2-locus might be an attractive tool due to its specificity for CD8^+^ cDC1 and could help to work out developmental and functional differences between CD8^+^ vs. CD103^+^ cDC1.

## Data Availability Statement

Sequence data that support the findings of this study have been deposited with the primary accession code GSE98235 at https://www.ncbi.nlm.nih.gov/geo/query/acc.cgi?acc=GSE98235. The other data that support the findings of this study are available from the corresponding author upon request.

## Author Contributions

MZ, TU, Y-LC, CR, CB, LG, and CS: conducted experiments; JS: performed sequencing analysis; DB and VB: provided adoptive transfer models and designed experiments; TB and SS: designed experiments and wrote the paper.

### Conflict of Interest Statement

The authors declare that the research was conducted in the absence of any commercial or financial relationships that could be construed as a potential conflict of interest.
