# Supplementary material for: Expression of the Phosphatase Ppef2 Controls Survival and Function of CD8+ Dendritic Cells
Source: Front Immunol. 2019 Feb 12;10:222. doi: 10.3389/fimmu.2019.00222 (PMC6379467; doi:10.3389/fimmu.2019.00222)
Supplement: Supplementary file 1 [file Data_Sheet_1.docx]

Supplementary Material

Expression Of The Phosphatase Ppef2 Controls Survival And Function Of CD8^+^ Dendritic Cells

**Markus Zwick, Thomas Ulas, Yi-Li Cho, Christine Ried, Leonie Grosse, Charlotte Simon, Caroline Bernhard, Dirk H. Busch, Joachim L. Schultze, Veit R. Buchholz, Susanne Stutte, Thomas Brocker^*^**

*** Correspondence:**

Corresponding Author: Thomas Brocker,

E-mail: tbrocker@med.uni-muenchen.de

Co-Corresponding Author: Susanne Stutte

E-mail: susanne.stutte@med.uni-muenchen.de

# Supplementary Data

Supplementary Material should be uploaded separately on submission. Please include any supplementary data, figures and/or tables. All supplementary files are deposited to FigShare for permanent storage and receive a DOI.

Supplementary material is not typeset so please ensure that all information is clearly presented, the appropriate caption is included in the file and not in the manuscript, and that the style conforms to the rest of the article.

# Supplementary Figures and Tables

For more information on Supplementary Material and for details on the different file types accepted, please see [here](http://home.frontiersin.org/about/author-guidelines#SupplementaryMaterial). Figures, tables, and images will be published under a Creative Commons CC-BY licence and permission must be obtained for use of copyrighted material from other sources (including re-published/adapted/modified/partial figures and images from the internet). It is the responsibility of the authors to acquire the licenses, to follow any citation instructions requested by third-party rights holders, and cover any supplementary charges.

## Supplementary Figures

**Supplementary Figure 1. Flow cytometry gating strategies for the identification of different cell types used in this study. (a)** Identification of DCs without (top panel) or with (lower panel) T and B cell depletion with anti-CD3ε and anti-CD19-coated microbeads from spleens. (**b**) Identification of CD4^+^ and CD8^+^ T cells, (**c**) B cells and (**d**) blood monocytes, (**e**) DC-precursors in spleen and bone marrow.


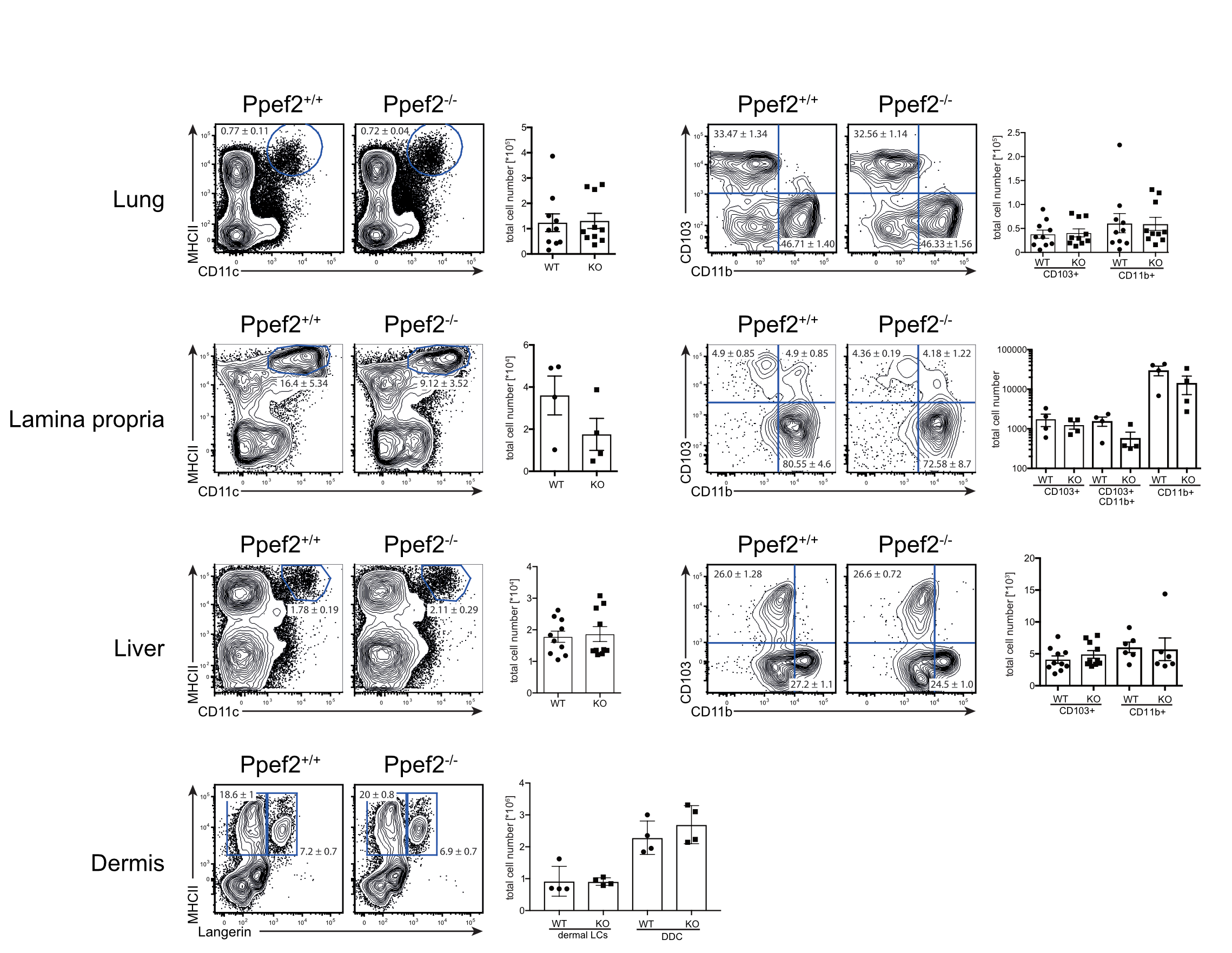


**Supplementary Figure 2. Percentage and total numbers of DC subsets are not altered in non-lymphoid organs of Ppef2^-/-^ mice.**

DCs of lung, lamina propria, skin and liver were identified as viable CD45^+^ CD11c^+^ MHCII^+^ and further distinguished by their expression of CD103 and CD11b. Shown are representative FACS-plots of pooled data (n=10) with the percentages of DCs ± SEM and the corresponding total numbers in the bar graphs next to the FACS-plots. Statistical analysis was performed using Student’s *t*-test. All p-values were above 0.05.

**Supplemental Figure 3. Phenotyping of hematopoietic cells does not show abnormalities.**

Spleens of Ppef2^+/+^ and Ppef2^-/-^mice were analyzed by the Centre d‘Immunophénomique (CIPHE) for various cell populations by flow cytometry (n=6 mice per group). The marker used for the identification of every population is listed in Suppl. Table 1. (**a**) Hierarchical clustering (HC) analysis of all 56 parameters tested in two different staining panels. Dataset was therefore imported into TIGR MultiExperiment Viewer (TMeV), normalized based on the median value of each row and reduced based on the SD of the values of the given row. A hierarchical clustering tree was built based on these signal intensities (Euclidean Complete). (**b**) Radar plot of the frequencies of different (sub-) populations (as percent of total or percent of parent, indicated in squared brackets) and their corresponding total cell numbers (**c**).

**Supplemental table 1.** Markers used to identify the subsets shown in Suppl. Fig. 3.

| **dump populations** | **marker** |
| --- | --- |
| Eosinophils | CD5- CD19- CD11b+ MHCII- SSChi |
| Neutrophils | CD5- CD19- Eosino- Macro- CD11b+ Ly6Cint |
| Monocytes | CD11b+ Ly6G- Ly6Chi |
| pDC | CD5- CD19- Eosino- Macro- Mono- CD11clo CD317+ CD45R+ Ly6C+ |
| Macrophages | CD11bint Ly6G- Ly6C- F4/80hi |
|  |  |
|  |  |
| **population** | **marker** |
| Eosinophils (Panel 4) | CD5- CD19- CD11b+ MHCII- SSChi |
| Infl. DCs | CD5- CD19- Eosino- Macro- Mono- pDC- CD11c+ MHCII+ CD64+ |
| Stage I (% pDC) | CD5- CD19- Eosino- Macro- Mono- CD11clo CD317+ CD45R+ Ly6C+ CD4- CD8- |
| gd T cells | CD161- Ly6G- CD317- CD5+ CD3+ TCRd+ |
| CD4 shedding [CD4 CD44-CD62L- (Shedding)] | CD161- Ly6G- CD317- CD5+ CD3+ TCRd- CD4+ CD44- CD62L- |
| Eosinophils (Panel 1) | CD11b+ Ly6G- Ly6Clo SSChi |
| CD4 Naive | CD161- Ly6G- CD317- CD5+ CD3+ TCRd- CD4+ CD44- CD62L+ |
| Cellularity | - |
| B1 B cells | CD161- Ly6G- CD317- CD19+ MHCII+ CD5+ |
| Xcr1 cDCs | CD5- CD19- Eosino- Macro- Mono- pDC- CD11c+ MHCII+ CD64- CD24+ CD172a- CD8a+ |
| IgDlo B2 B cells | CD161- Ly6G- CD317- CD19+ MHCII+ CD5- IgDlo |
| CD8 naive | CD161- Ly6G- CD317- CD5+ CD3+ TCRd- CD8+ CD44- CD62L+ |
| gd Naive | CD161- Ly6G- CD317- CD5+ CD3+ TCRd+ CD44- CD62L+ |
| CD4- CD8+ (%pDCs) | CD5- CD19- Eosino- Macro- Mono- CD11clo CD317+ CD45R+ Ly6C+ CD4- CD8+ |
|  |  |
| **population** | **marker** |
| CD8 shedding [CD8 CD44-CD62L- (Shedding)] | CD161- Ly6G- CD317- CD5+ CD3+ TCRd- CD8+ CD44- CD62L- |
| gd shedding [gd cd44-cd62l- (Shedding)] | CD161- Ly6G- CD317- CD5+ CD3+ TCRd+ CD44- CD62L- |
| NKT | CD161+ Ly6G- CD317- CD5+ |
| IgDhi B2 B cells | CD161- Ly6G- CD317- CD19+ MHCII+ CD5- IgDhi |
| CD8 | CD161- Ly6G- CD317- CD5+ CD3+ TCRd- CD8+ |
| CD4 CM | CD161- Ly6G- CD317- CD5+ CD3+ TCRd- CD4+ CD44+ CD62L+ |
| NK CD11b+Ly6C+ | CD161+ Ly6G- CD317- CD5- CD11b+ Ly6C+ |
| CD4 EM | CD161- Ly6G- CD317- CD5+ CD3+ TCRd- CD4+ CD44+ CD62L- |
| NK | CD161+ Ly6G- CD317- CD5- |
| CD8 CM | CD161- Ly6G- CD317- CD5+ CD3+ TCRd- CD8+ CD44+ CD62L+ |
| B cells | CD161- Ly6G- CD317- CD19+ MHCII+ |
| Viability | - |
| B2 B cells | CD161- Ly6G- CD317- CD19+ MHCII+ CD5- |
| ab T cells | CD161- Ly6G- CD317- CD5+ CD3+ TCRd- |
| CD4 | CD161- Ly6G- CD317- CD5+ CD3+ TCRd- CD4+ |
| CD11b+ DCs | CD5- CD19- Eosino- Macro- Mono- pDC- CD11c+ MHCII+ CD64- CD24+/- CD172a+ CD11b+ |
| Imm CD11b+ DCs | CD5- CD19- Eosino- Macro- Mono- pDC- CD11c+ MHCII+ CD64- CD24+/- CD172a+ CD11b+ CD117+ |
| Imm Xcr1 DCs | CD5- CD19- Eosino- Macro- Mono- pDC- CD11c+ MHCIIlo CD64- CD24+ CD172a- CD8a+ CD117+ |
| gd CM | CD161- Ly6G- CD317- CD5+ CD3+ TCRd+ CD44+ CD62L+ |
| CD11b-type DC | CD11b- Ly6G- CD11b- CD317- CD11c+ MHCII+ CD11b+ |
|  |  |
|  |  |
| **population** | **marker** |
| NK CD11b-Ly6C+ | CD161+ Ly6G- CD317- CD5- CD11b- Ly6C+ |
| Stage III (%pDCs) | CD5- CD19- Eosino- Macro- Mono- CD11clo CD317+ CD45R+ Ly6C+ CD4+ CD8- |
| gd EM | CD161- Ly6G- CD317- CD5+ CD3+ TCRd+ CD44+ CD62L- |
| Stage II (%pDC) | CD5- CD19- Eosino- Macro- Mono- CD11clo CD317+ CD45R+ Ly6C+ CD4+ CD8+ |
| CD8 EM | CD161- Ly6G- CD317- CD5+ CD3+ TCRd- CD8+ CD44+ CD62L- |
| Mat Xcr1 DC | CD5- CD19- Eosino- Macro- Mono- pDC- CD11c+ MHCIIlo CD64- CD24+ CD172a- CD8a+ CD117- |
| Mat CD11b+ DC | CD5- CD19- Eosino- Macro- Mono- pDC- CD11c+ MHCII+ CD64- CD24+/- CD172a+ CD11b+ CD117- |
| Xcr1 DC | CD5- CD19- Eosino- Macro- Mono- pDC- CD11c+ MHCII+ CD64- CD24+ CD172a- CD8a+ |
| Macrophages | CD5- CD19- CD24+ CD172a+ CD64+ |
| cDC (Panel 4) | CD5- CD19- Eosino- Macro- Mono- pDC- CD11c+ MHCII+ |
| Neutrophils (Panel 1) | CD11b+ Ly6G+ |
| Neutrophils (Panel 4) | CD5- CD19- Eosino- Macro- CD11b+ Ly6Cint |
| Monocytes | CD5- CD19- Eosino- Macro- CD11b+ Ly6C+ |
| NK CD11b-Ly6C- | CD161+ Ly6G- CD317- CD5- CD11b- Ly6C+ |
| Monocytes | CD11b+ Ly6G- Ly6Chi |
| pDC (Panel 1) | CD11b- Ly6G- Ly6Chi CD317+ |
| pDC (Panel 4) | CD5- CD19- Eosino- Macro- Mono- CD11clo CD317+ CD45R+ Ly6C+ |
| Other Mac | CD11bhi Ly6G- Ly6C- F4/80hi |
| RP Macrophages | CD11bint Ly6G- Ly6C- F4/80hi |
